# Supplementary material for: Early Cambrian origin of the shelf sediment mixed layer
Source: Nat Commun. 2018 May 15;9:1909. doi: 10.1038/s41467-018-04311-8 (PMC5953921; doi:10.1038/s41467-018-04311-8)
Supplement: Supplementary file 1 — Supplementary Information [file 41467_2018_4311_MOESM1_ESM.pdf]

Early Cambrian origin of the shelf sediment mixed layer

Gougeon et al.

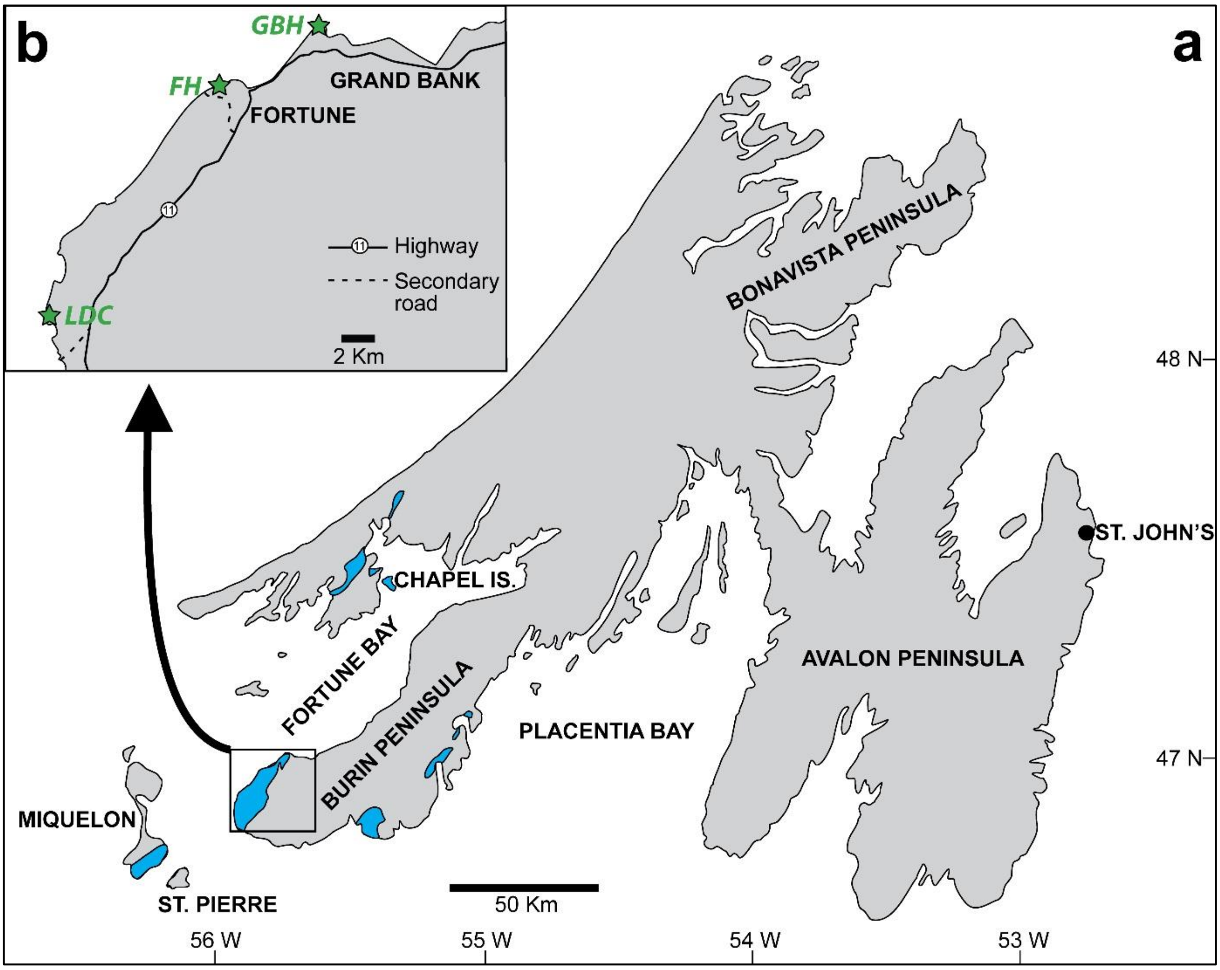

Supplementary Figure 1 | Location map of the study area. (**a**) General map of eastern Newfoundland showing location of Burin Peninsula. Important Cambrian outcrops are figured in blue color. (**b**) Detailed map showing location of the outcrops studied. GBH = Grand Bank Head; FH = Fortune Head; LDC = Little Dantzic Cove.

Sample in Fig. 2	Total area	Bioturbated area	Bioturbation percentage	BI
**2e**	4151746	4151746	100.0%	6
**2d**	2048936	2048936	100.0%	6
**2c**	6183546	5896821	95.4%	5
**2b**	1987289	418440	21.1%	2
**2a**	3446019	9245	0.3%	0

Supplementary Table 1 | Summary of the bioturbation values obtained after using ImageJ for the five samples of Fig. 2.

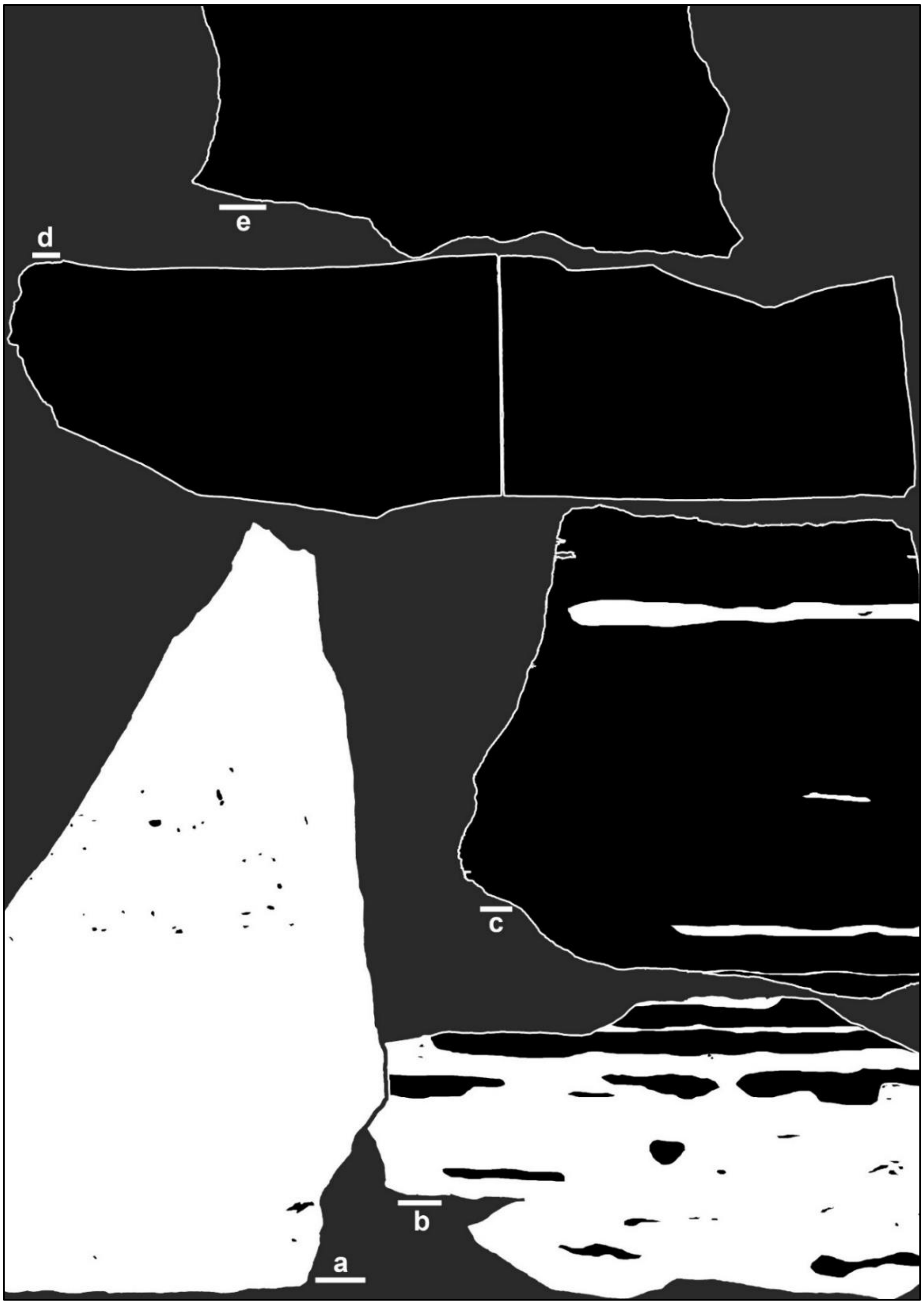

Supplementary Figure 2 | Alternative view of Fig. 2 revealing the bioturbated zones (in black) of the five samples. After this step, each sample has been processed using ImageJ in order to reveal their bioturbation percentage. Scale bars are 1 cm long.

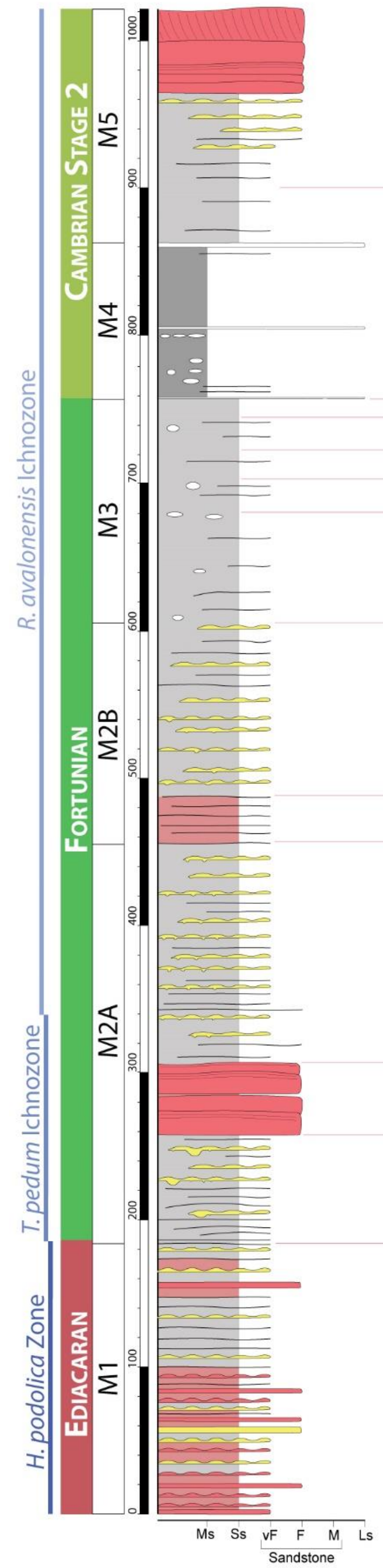

Supplementary Figure 3 | Stratigraphic column showing the stratigraphic intervals corresponding to the polished samples shown in Supplementary Notes 1-7.

Supplementary Note 1

Interval **1 -** Lower Fortunian, member 2A, from base of member 2A to base of red beds

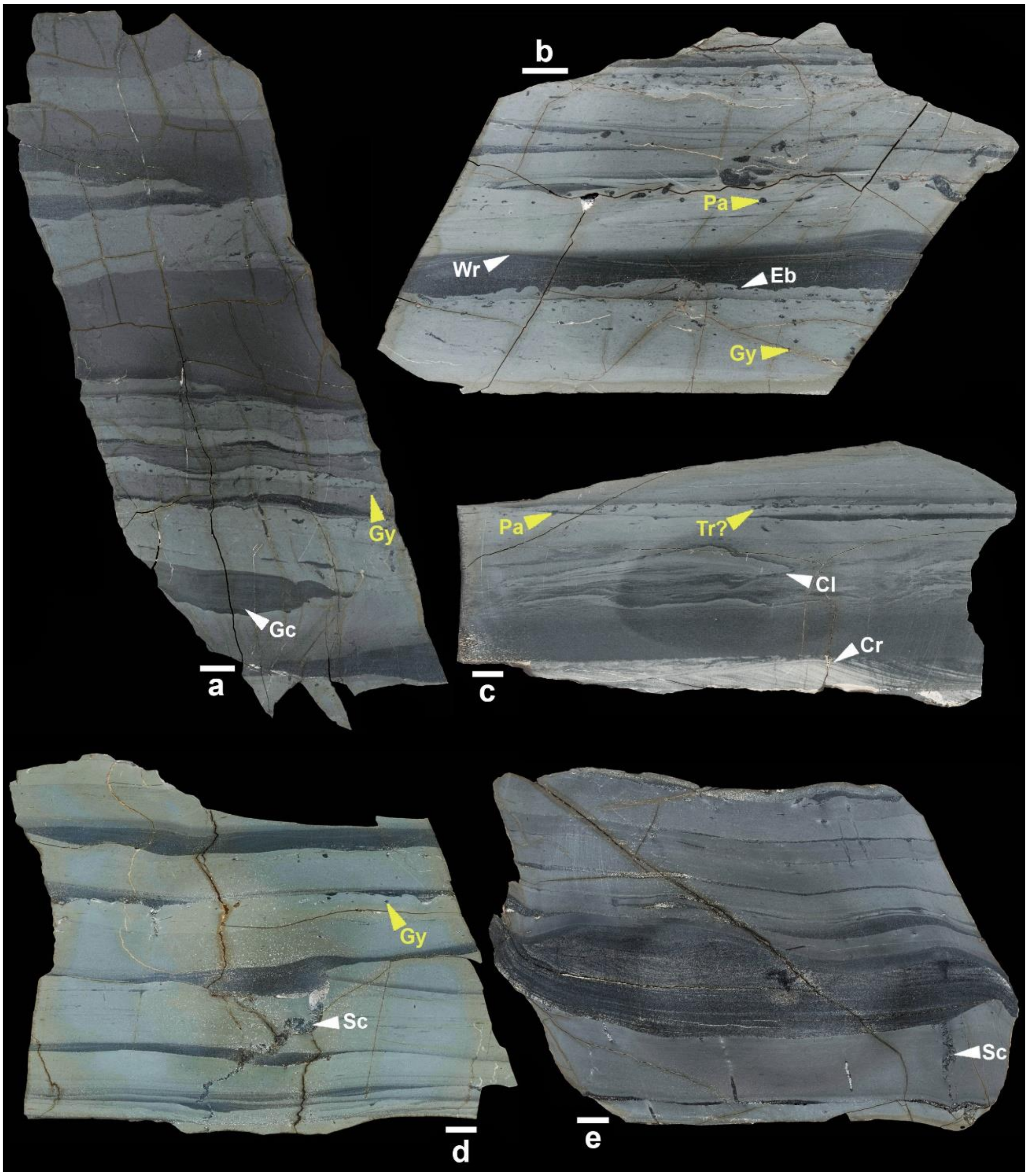

Supplementary Figure 4 | Polished slabs from the lower Fortunian of Fortune Head (187-272 m), showing heterolithics and low bioturbation levels. Sedimentary features: Cl = convolute lamination; Cr = current ripple; Eb = erosive base; Gc = gutter cast; Sc = syneresis crack; Wr = wave ripple. Trace fossils: Gy = *Gyrolithes*; Pa = *Palaeophycus*; Tr = *Treptichnus*. Scale bars are 1 cm long.

Sample in Supplementary Fig. 4	Total area	Bioturbated area	Bioturbation percentage	BI
**S4a**	2818612	11579	0.4%	0
**S4b**	3421792	52856	1.5%	1
**S4c**	3097648	19687	0.6%	0
**S4d**	4805348	11135	0.2%	0
**S4e**	4748313	27634	0.6%	0

Supplementary Table 2 | Summary of the bioturbation values obtained from ImageJ processing for the five samples of the Supplementary Figure 4.

Supplementary Figure 4 illustrates five samples from the Fortune Head outcrop, just above the base of the Cambrian GSSP. All samples show exquisite preservation of heterolithic lamina or very thin beds represented by siltstone and very-fine to fine-grained sandstone. Sand lamina are highly consistent laterally showing little variation in thickness (Supplementary Fig. 4d, e), indicating negligible to very minor biogenic disturbance. No background mottling typical of a sediment mixed layer is apparent, each bed showing sharp contrast in color. Slabs illustrate nicely preserved sedimentary features, such as wave (Supplementary Fig. 4b) and current ripples (Supplementary Fig. 4c), convolute lamination (Supplementary Fig. 4c), sharp erosive bases of sandstone layers (Supplementary Fig. 4b), pot/gutter cast (Supplementary Fig. 4a) and syneresis cracks (Supplementary Fig. 4d, e). Trace fossils are represented by pristine, sharp-walled and passively filled open burrows that only minimally disturb the sedimentary fabric. Bioturbation Index ranges from 0 to 1 (Supplementary Table 2) and burrows are represented by shallow-tier *Gyrolithes*, tiny *Palaeophycus* and treptichnids emplaced in cohesive substrates.

<u>Supplementary Note 2</u>

<u>Interval **2** - Lower Fortunian, member 2A, from top of red beds to base of member 2B</u>

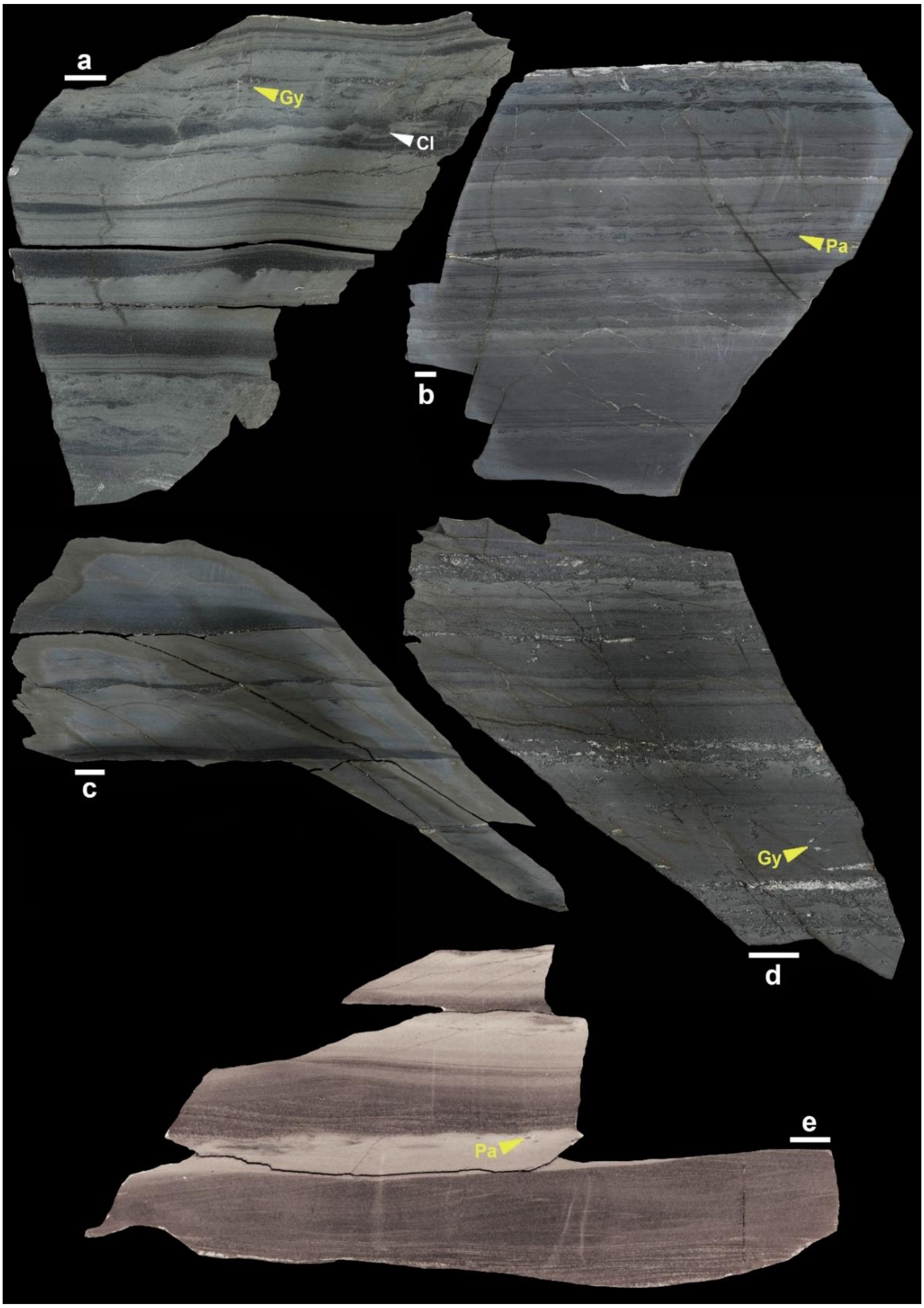

Supplementary Figure 5 | Polished slabs from the lower Fortunian of Fortune Head (306-454 m), showing heterolithics and low bioturbation levels. Sedimentary features: Cl = convolute lamination. Trace fossils: Gy = *Gyrolithes*; Pa = *Palaeophycus*. Scale bars are 1 cm long.

Sample in Supplementary Fig. 5	Total area	Bioturbated area	Bioturbation percentage	BI
**S5a**	4913941	215646	4.4%	1
**S5b**	189942122	13575893	7.1%	2
**S5c**	2385140	668	0.0%	0
**S5d**	3957741	324552	8.2%	2
**S5e**	2056382	893	0.0%	0

Supplementary Table 3 | Summary of the bioturbation values obtained from ImageJ processing for the five samples of the Supplementary Figure 5.

Supplementary Figure 5 corresponds to the Fortune Head outcrop and displays heterolithic lamina and very thin beds of siltstone and very fine- to fine-grained sandstone, only locally reaching medium to coarse-grained sandstone (Supplementary Fig. 5d). Beds and lamina are still laterally continuous (Supplementary Fig. 5a, b, d), revealing a low bioturbation index (maximum BI=2). Convolute lamination (Supplementary Fig. 5a) is common locally. Burrows are preferentially represented by unlined, open structures, which are typically infilled by coarse-grained sand (Supplementary Fig. 5d), representing penetration in firm substrates. *Gyrolithes* and tiny *Palaeophycus* are present.

<u>Supplementary Note 3</u>

<u>Interval **3** - Middle Fortunian, member 2B, from top of red beds to base of member 3</u>

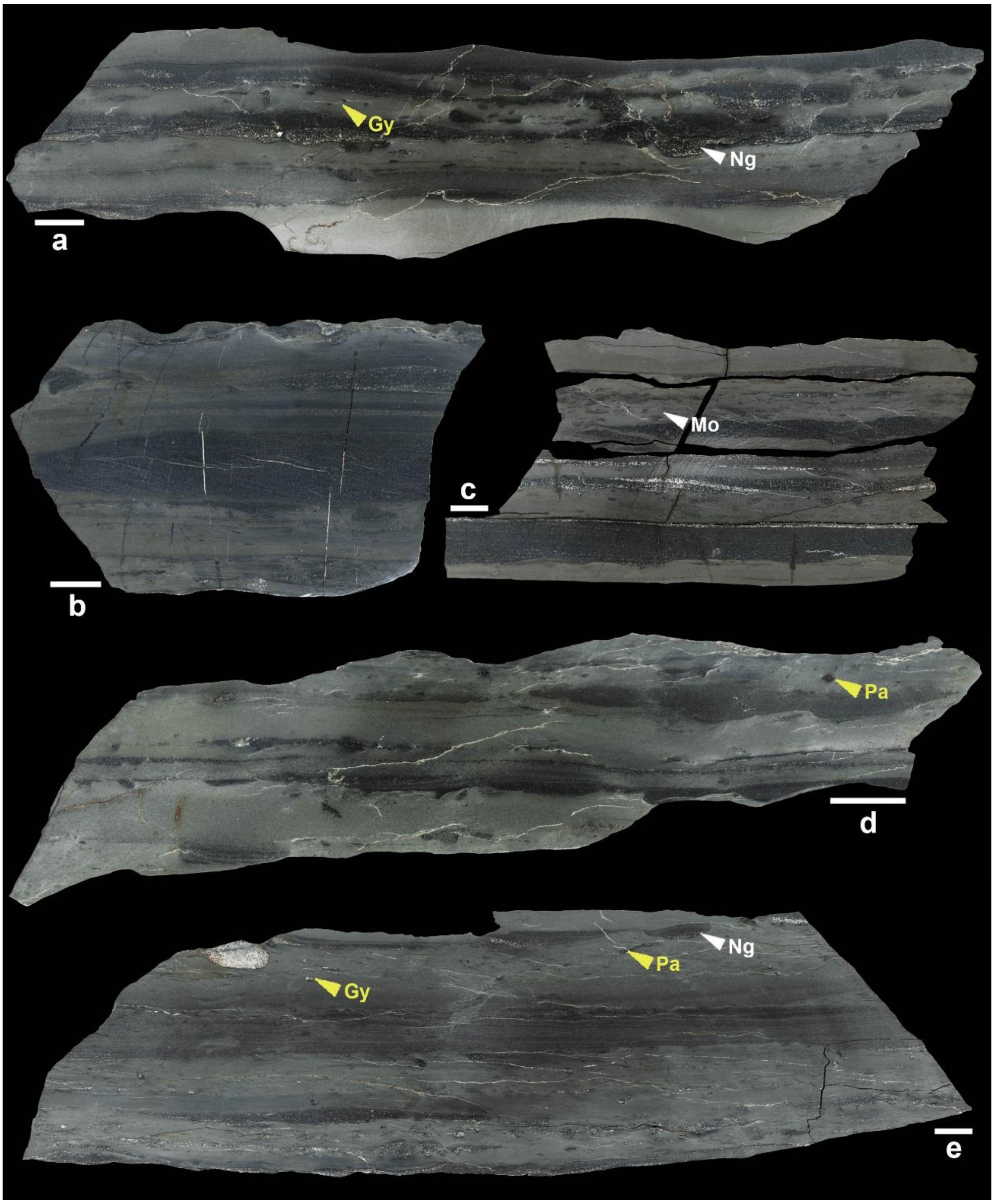

Supplementary Figure 6 | Polished slabs from the middle Fortunian of Fortune Head (a, c) and Grand Bank Head (b, d, e) (463-612 m), showing heterolithics and low bioturbation levels. Sedimentary features: Mo = incipient mottling texture; Ng = normal grading in sandy beds. Trace fossils: Gy = *Gyrolithes*; Pa = *Palaeophycus*. Scale bars are 1 cm long.

Sample in Supplementary Fig. 6	Total area	Bioturbated area	Bioturbation percentage	BI
**S6a**	1438875	59343	4.1%	1
**S6b**	3948167	115602	2.9%	1
**S6c**	2960886	236072	8.0%	2
**S6d**	1412657	106906	7.6%	2
**S6e**	1834302	182117	9.9%	2

Supplementary Table 4 | Summary of the bioturbation values obtained from ImageJ processing for the five samples of the Supplementary Figure 6.

Supplementary Figure 6 illustrates samples from Fortune Head and Grand Bank Head, which are characterized by sharp, isolated, shallow-tier burrows emplaced in firmgrounds. However, thin lamina of sharp-based, very fine- to fine-grained sandstone are locally disturbed by slightly more intense bioturbation (Supplementary Table 4). Individual lamina are still well-preserved, but less persistent laterally (Supplementary Fig. 6a, d). In coarser-grained sandstone beds, normal grading from coarse- to fine-grained sandstone is common (Supplementary Fig. 6a, e). Unlined open burrows are filled with medium- to coarse-grained sand. Burrow diameter is also larger than in trace fossils from underlying strata. Trace fossils include *Gyrolithes*, *Palaeophycus* and indistinct burrows. Incipient mottling textures are locally present (Supplementary Fig. 6c), indicating spots of increased bioturbation intensity conducive to more significant disturbance of sandstone lamination and a random orientation of coarse sand grains. However, maximum bioturbation index is still similar to the one recorded in underlying strata (BI=2).

<u>Supplementary Note 4</u>

<u>Interval **4** - Upper Fortunian, from base to middle of member 3</u>

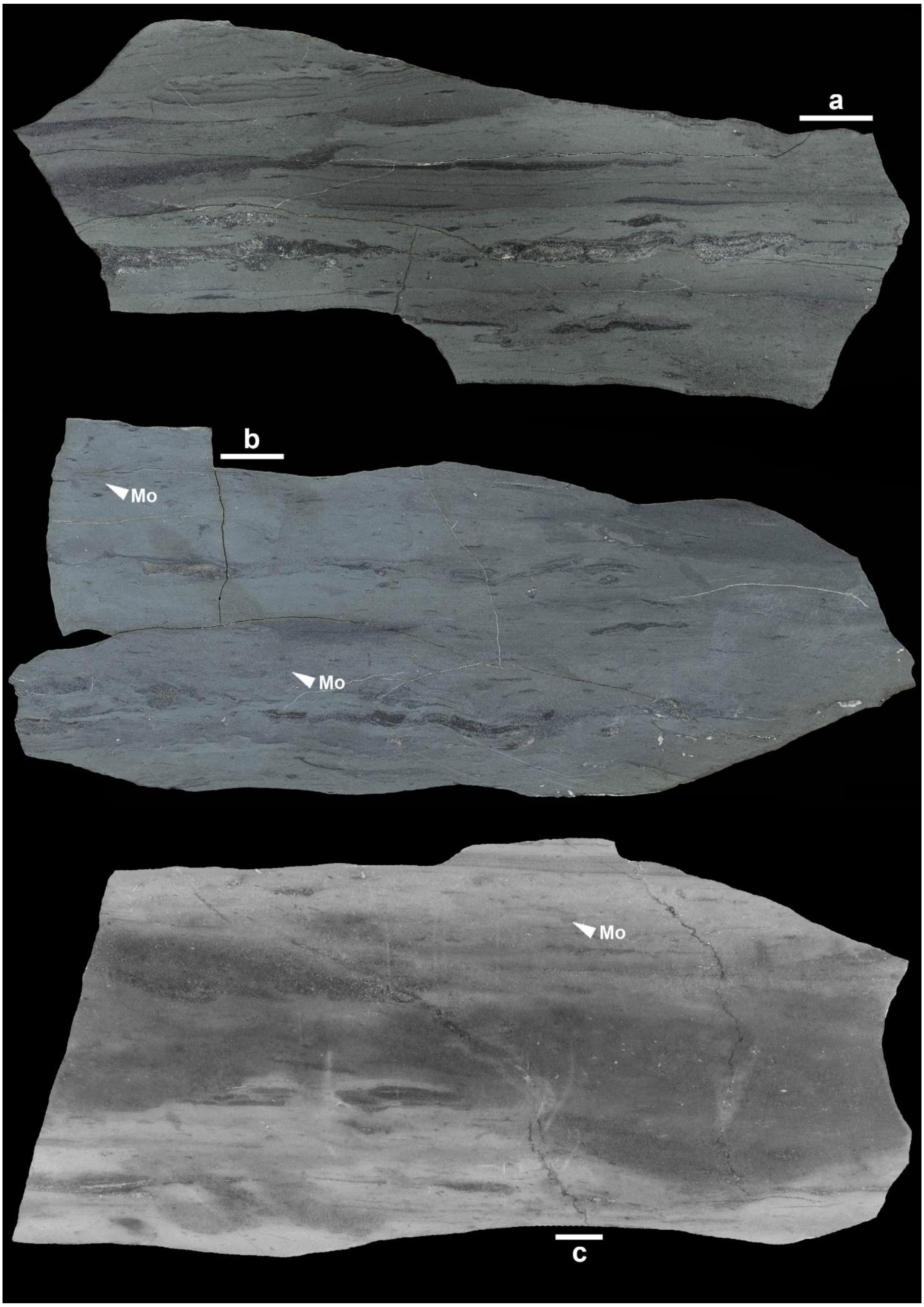

Supplementary Figure 7 | Polished slabs from the upper Fortunian of Little Dantzic Cove (612-685 m), showing mottling and medium bioturbation levels.

Sedimentary features: Mo = incipient mottling texture. Scale bars are 1 cm long.

Sample in Supplementary Fig. 7	Total area	Bioturbated area	Bioturbation percentage	BI
**S7a**	2398060	990684	41.3%	3
**S7b**	2690953	2336840	86.8%	4
**S7c**	3137042	1465374	46.7%	3

Supplementary Table 5 | Summary of the bioturbation values obtained from ImageJ processing for the three samples of the Supplementary Figure 7.

These three samples correspond to the Little Dantzic Cove outcrop and indicate a progressive increase of biogenic disturbance from members 2 to 3. Beds are typically composed of very-fine to medium-grained sandstone (Supplementary Fig. 7a). A few, distinct sandstone lamina are recorded, but overall sandy lamina tend to be more intensively disturbed by bioturbation (Supplementary Fig. 7a, b). As a result, sandstone beds are laterally discontinuous, showing more extreme thickness variations (Supplementary Fig. 7a, b). Discrete, well-defined burrows become rarer, therefore complicating ichnotaxonomic determination; mottled background clearly occurs for the first time (Supplementary Fig. 7b) resulting in higher degrees of bioturbation (Supplementary Table 5).

Supplementary Note 5

Interval **5** - Upper Fortunian, top of member 3

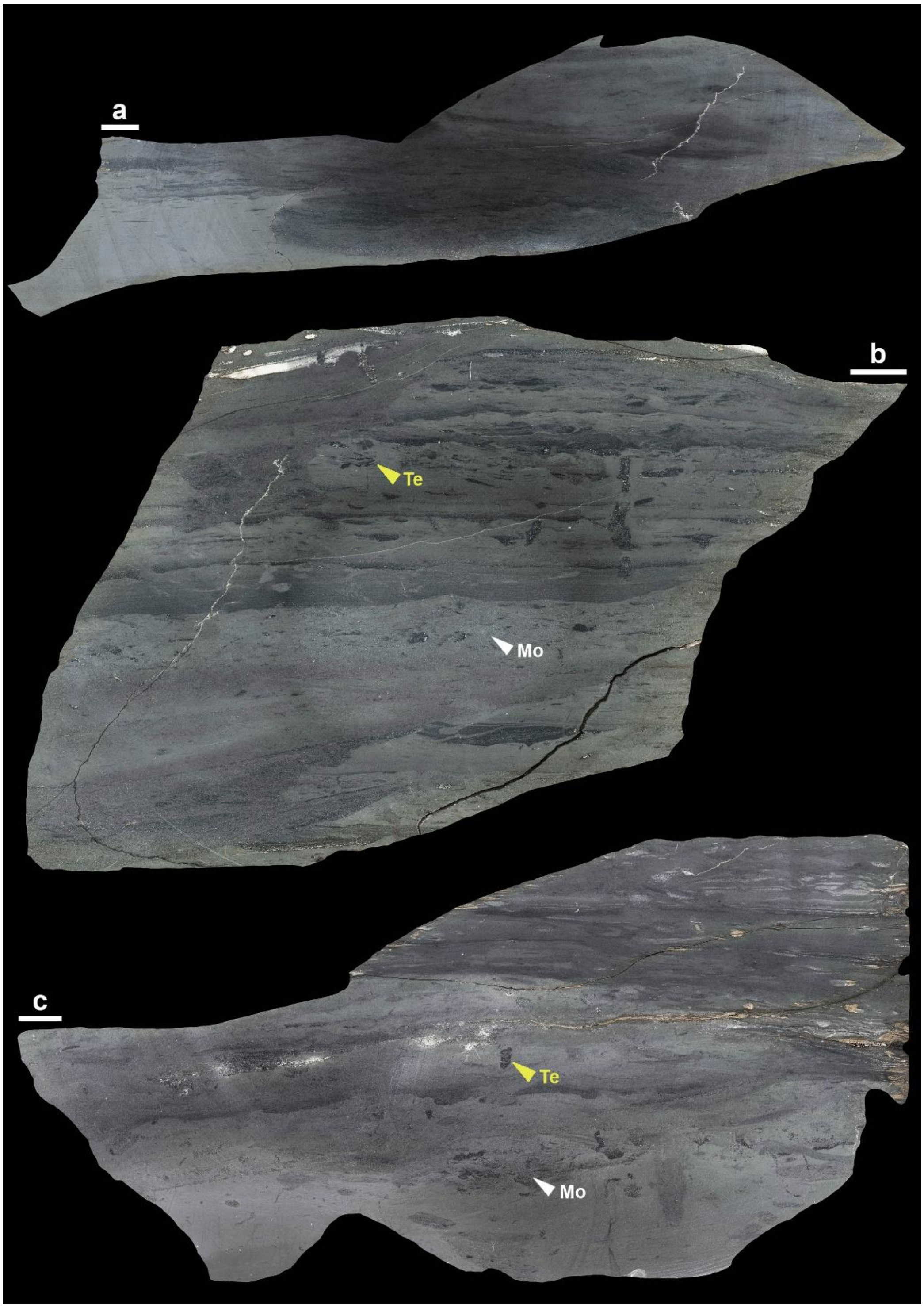

Supplementary Figure 8 | Polished slabs from the upper Fortunian of Little Dantzic Cove (704-724 m), showing mottling and medium to high bioturbation levels. Sedimentary features: Mo = incipient mottling texture. Trace fossils: Te = *Teichichnus*. Scale bars are 1 cm long.

Sample in Supplementary Fig. 8	Total area	Bioturbated area	Bioturbation percentage	BI
**S8a**	1222023	1021333	83.6%	4
**S8b**	3367549	1979156	58.8%	3
**S8c**	2679588	2439685	91.0%	5

Supplementary Table 6 | Summary of the bioturbation values obtained from ImageJ processing for the three samples of the Supplementary Figure 8.

Supplementary Figure 8 illustrates samples from Little Dantzic Cove. Siltstone to very fine- and fine-grained sandstone layers are dominant. The classic preservation style (well-defined heterolithic bedding) of underlying strata is still locally present (top of Supplementary Fig. 8b). However, much higher biogenic disturbance become dominant (Supplementary Table 6), as revealed by the abundant intervals showing mottled textures (Supplementary Fig. 8b, c). Locally vertical burrows filled with medium-grained sand are present (Supplementary Fig. 8b). This is accompanied by a change in ichnotaxonomic composition with the first appearance of the vertical spreiten feeding trace *Teichichnus* (Supplementary Fig. 8b, c). Supplementary Figure 8b displays two intervals of burrowing intensity that depend on the rates of sedimentation/erosion. When the sedimentation rate is high (top of Supplementary Fig. 8b), opportunistic colonizers of the substrate can disturb the laminated sediment. The brief colonization window prevents the establishment of a mature equilibrium community (as it is shown in the case of a sediment mixed layer). On the other hand, the base of Supplementary Figure 8b displays slower sedimentation rates which allow an extended colonization window for the infauna to process the sediment by repeated reworking and multiple burrow overlap. These processes result in the destruction of the primary sedimentary fabric. In that case, intense biogenic activity is conducive to the formation of a sediment mixed layer during fair-weather times.

Supplementary Note 6

Interval **6** - Uppermost Fortunian, top of member 3

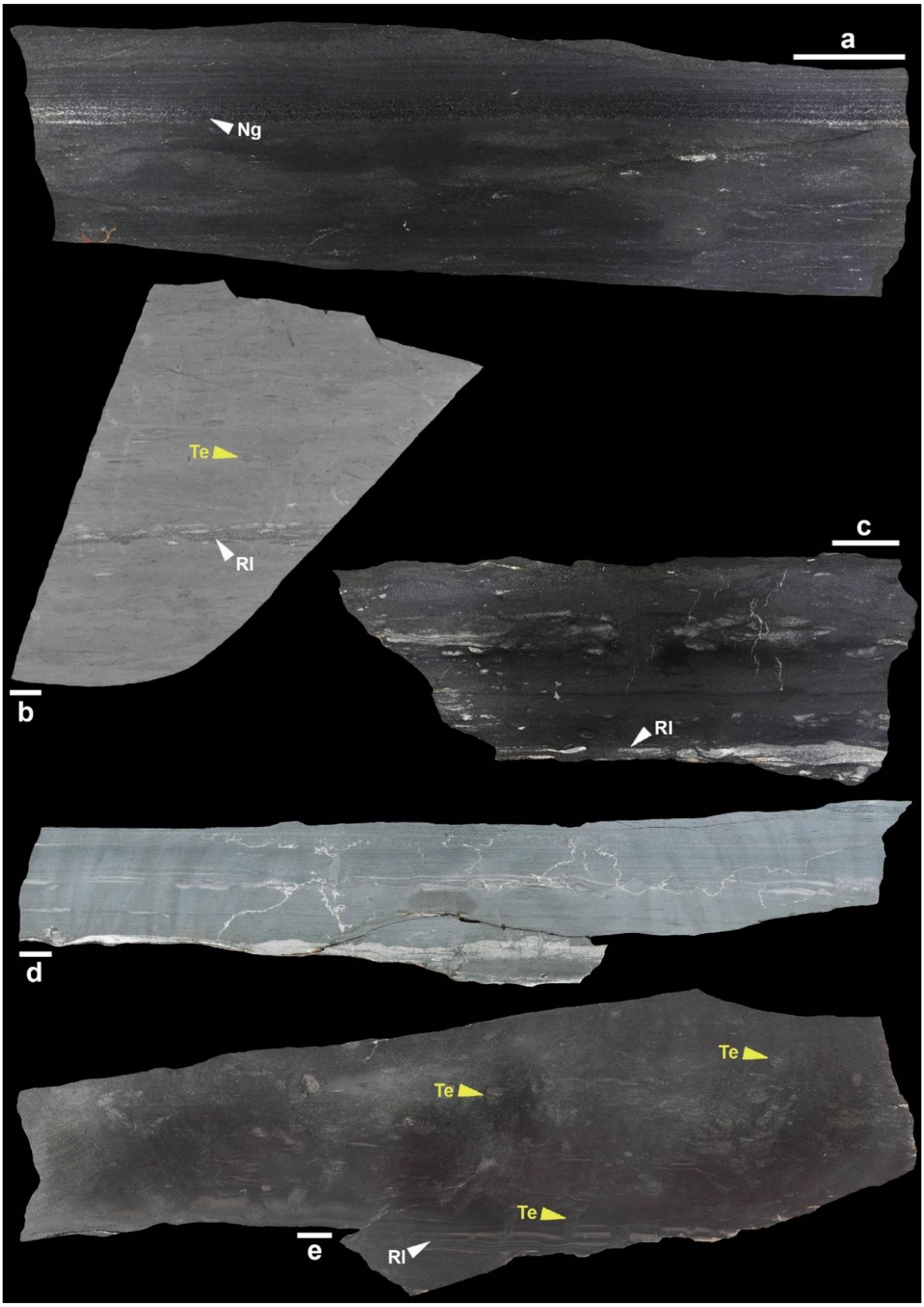

Supplementary Figure 9 | Polished slabs from the upper Fortunian of Little Dantzic Cove (725-745 m), showing mottling and medium to high bioturbation levels. Sedimentary features: Ng = normal grading of sandy beds; Rl = remnant of primary lamination/bedding. Trace fossils: Te = *Teichichnus*. Scale bars are 1 cm long.

Sample in Supplementary Fig. 9	Total area	Bioturbated area	Bioturbation percentage	BI
**S9a**	2098134	1062053	50.6%	3
**S9b**	3841377	3705226	96.5%	5
**S9c**	2484774	2109656	84.9%	4
**S9d**	1194910	806672	67.5%	4
**S9e**	1900248	1771513	93.2%	5

Supplementary Table 7 | Summary of the bioturbation values obtained from ImageJ processing for the five samples of the Supplementary Figure 9.

Supplementary Figure 9 displays slabs from Little Dantzic Cove. Three samples (Supplementary Fig. 9a, c, e) come from red beds at the top of member 3, which show the classic alternation of green and grey siltstone facies. Red beds from member 2 of the Chapel Island Formation (i.e. from the middle of member 2A to the base of member 2B) never show any evidence of bioturbation. In identical facies from the uppermost part of member 3, absence of bioturbation is the exception rather than the rule, as shown by the local presence of unbioturbated, normally graded, medium- to fine-grained sandstone encased in siltstone and sandy siltstone (Supplementary Fig. 9a). However, the most representative samples from these red beds, but also green/grey slabs consist of sharp-based sandstone tempestites having bioturbated tops and encased in bioturbated fair-weather mudstone (Supplementary Fig. 9b, e). Some of these beds show important reworking of sediment (BI=5, Supplementary Table 7) by mid-tier *Teichichnus* disturbing thin sandstone lamina or cross-cutting a mottled background fabric. In many cases, intense bioturbation evidenced by mottled background results in preservation of only remnants of the primary lamination (Supplementary Fig. 9b, e).

Supplementary Note 7

Interval 7 - Lower Cambrian Stage 2, members 4 and 5

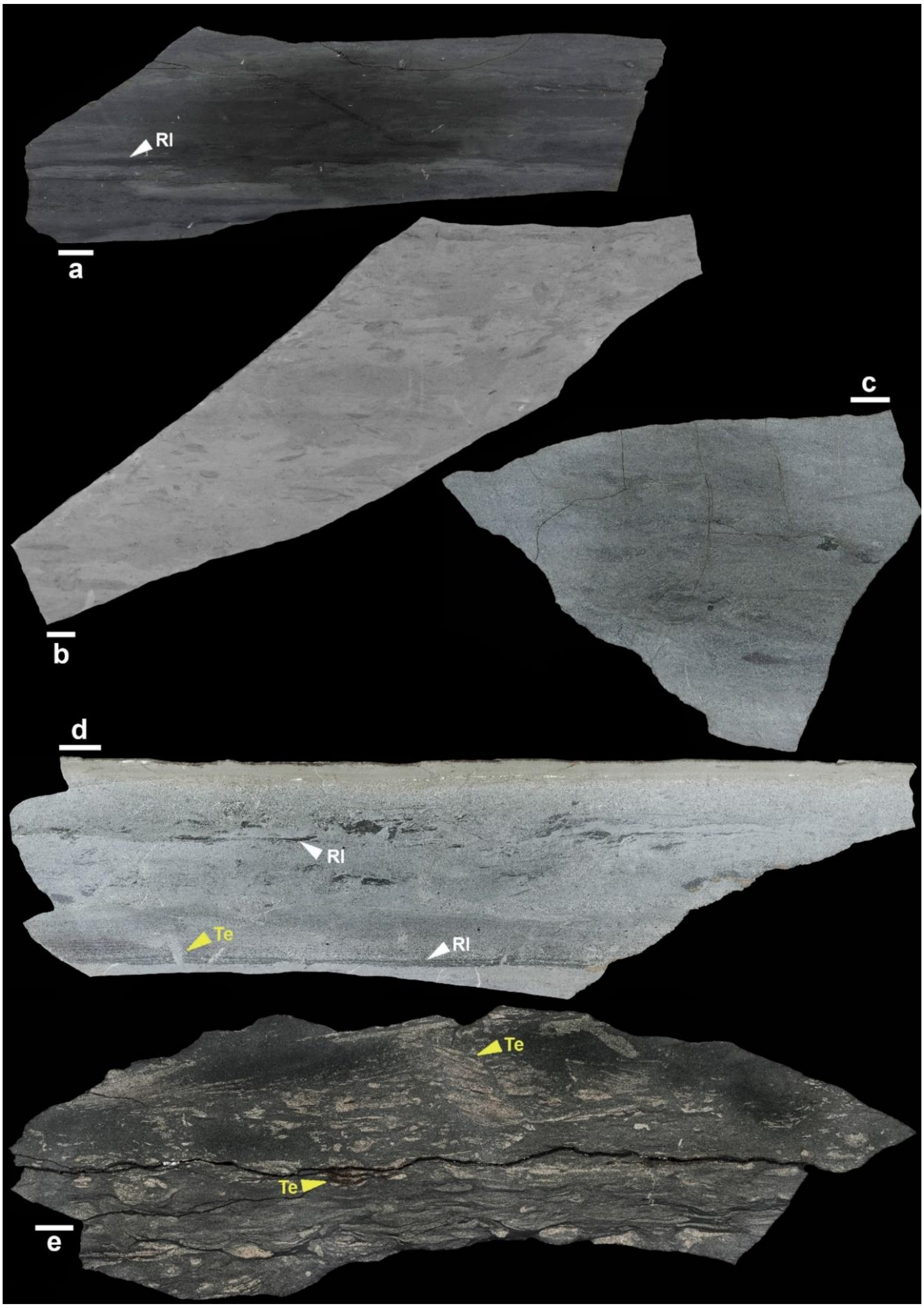

Supplementary Figure 10 | Polished slabs from the lower Cambrian Stage 2 of Little Dantzic Cove (766-899 m), showing mottling and high bioturbation levels. Sedimentary features: Rl = remnant of primary lamination/bedding. Trace fossils: Te = *Teichichnus*. Scale bars are 1 cm long.

Sample in Supplementary Fig. 10	Total area	Bioturbated area	Bioturbation percentage	BI
**S10a**	2078734	985761	47.4%	3
**S10b**	1958010	1958010	100.0%	6
**S10c**	3622254	3622254	100.0%	6
**S10d**	1737932	1368773	78.8%	4
**S10e**	1880815	1880815	100.0%	6

Supplementary Table 8 | Summary of the bioturbation values obtained from ImageJ processing for the five samples of the Supplementary Figure 10.

Supplementary Figure 10 illustrates samples from Little Dantzic Cove. The dominant style is characterized by intense bioturbation (maximum BI=6, Supplementary Table 8). Primary fabric is in most cases almost completely obliterated by mottled textures and no bedding or lamination is apparent (Supplementary Fig. 10b, c). Only the thickest tempestites are preserved, as evidenced by a sharp base, a coarser-grained, unbioturbated basal division and upper portions almost completely disturbed by bioturbation (Supplementary Fig. 10d). Mid-tier *Teichichnus* cross-cut the sandstone bed at the base below the mixed layer (into the transition layer), illustrating similarities with the vertical partitioning of the infaunal habitat in modern marine sediments. Medium-grained sand filled *Teichichnus* emplaced in the transition zone are seen cross-cutting extremely bioturbated background fabric indicative of the soupy substrate conditions of the mixed layer (Supplementary Fig. 10e). A few samples are characterized by mottling and laterally non-persistent sandstone and siltstone layers displaying thickness variations (Supplementary Fig. 10a). Overall, Cambrian Stage 2 strata in Little Dantzic Cove show the appearance of the mixed layer in the fossil record.
